# RACIAL/ETHNIC DISPARITIES IN MARITAL STATUS AND LIVING ARRANGEMENT TRANSITIONS AMONG US OLDER ADULTS

**DOI:** 10.1093/geroni/igad104.3763

**Published:** 2023-12-21

**Authors:** Xueqing Wang, Xuezhixing Zhang, Emma Zang

**Affiliations:** Princeton University, Princeton, New Jersey, United States; Yale University, New Haven, Connecticut, United States; Yale University, New Haven, Connecticut, United States

## Abstract

US older adults’ family life, including marital status and living arrangement, has become increasingly complex and diverse in recent decades. However, there is limited understanding of the average duration each racial/ethnic group typically spends in different marital statuses and living arrangements during older ages. Using data from the Health and Retirement Study 1992-2018, this research note investigates the racial/ethnic differences in the expected years to be spent at age 50 in each of the marital statuses (i.e., married, divorced, widowed, and never married) and living arrangements (i.e., living alone, living with spouse, living with non-spouses only, and nursing home. At age 50, Whites spend significantly more time married compared to Blacks and Hispanics, primarily due to the latter spending longer years divorced or never-married. Regarding living arrangements, Whites also spend notably more time living with spouse than Blacks and Hispanics, with the latter spending more time living only with non-spouses. These findings suggest that Whites have a distinct advantage in maintaining a stable, two-person family structure in late adulthood, while Blacks and Hispanics are more likely to spend their later years without their spouses. These patterns have broad implications for understanding persistent social inequality in late adulthood.

